# On the correctness of using two-dimensional representations in the analysis of cylindrical peg–hole insertion and withdrawal

**DOI:** 10.1098/rsos.221021

**Published:** 2023-08-30

**Authors:** Feiying Lan, Marco Castellani, Duc Truong Pham, Yongjing Wang

**Affiliations:** Department of Mechanical Engineering, University of Birmingham, Birmingham B15 2TT, UK

**Keywords:** peg–hole problem, geometrical equivalence, kinematic non-equivalence, simplification, jamming and wedging, remote compliance centre

## Abstract

The cylindrical peg–hole system is a popular model in the study of assembly and disassembly operations. The analysis of peg–hole systems is customarily performed using simplified two-dimensional representations, *viz*. a vertical sectional view. However, evidence that this simplification accurately represents the system is lacking. This paper investigates the correctness of using two-dimensional instead of three-dimensional models for peg–hole system analysis, studying their geometrical and kinematic equivalence. Geometrical equivalence implies the contact points between the peg and hole are on a vertical sectional view plane. Kinematic equivalence requires that the forces and torques acting on the peg lie in the same sectional plane. The analysis indicates that while geometrical equivalence can be proven, kinematic equivalence is in general not verified. The severity of the error introduced by the two-dimensional simplification depends on the geometrical configuration and kinematic parameters of the peg–hole system. The effects of kinematic non-equivalence on the boundary conditions of jamming and wedging are discussed. The results of the analysis show that a two-dimensional peg–hole model may give wrong predictions on jamming. Also, the extra lateral error of the three-dimensional model reduces the boundary condition and the possibility of peg–hole wedging.

## 1. Introduction

Remanufacturing is an important component of a circular economy. Its aim is to recover end-of-life products to like-new functional state, or the original manufacturing specifications [1,2]. Remanufacturing is quickly gaining attention due to its importance to environmental [3] and economic [4,5] sustainability.

Disassembly is the process of systematic removal of components and materials from end-of-life products [6]. It is the first step in the reprocessing of returned products, and to date is still largely performed manually. Automation of disassembly processes [7] is highly desirable due to the time-consuming, costly, and sometimes hazardous nature of disassembly tasks. It includes the solution of a number of fundamental generic tasks such as unscrewing, removal of pins from holes with small clearances, and separation of press-fit components. Amongst these tasks, the removal of peg-like components from holes is a common occurrence [8]. Robotic peg–hole assembly has been extensively studied in industrial manufacturing. For example, Qiao *et al.* [9] developed a chamferless peg–hole insertion strategy in which a six-component force sensor was used to identify the the relative position of the peg and the hole. Unten *et al.* [10] found that accurate relative positioning between the peg and hole could be determined from transient contact state. Thus, based on this understanding, Unten *et al.* [10] proposed an approach to perform precise peg–hole insertion by reproducing the transient contact state. So far, however, robotic disassembly has been the subject of little research [8,11].

Jamming and wedging are two common problems in peg–hole insertion and withdrawal operations. Jamming is the condition where the peg is not able to move further due to misplaced forces and torques. Wedging is the condition where the peg is stuck at a position due to geometric misplacement, and in severe cases no further movement is possible without damaging the parts [12]. Pioneering work on the avoidance of jamming and wedging was carried out by Whitney [12]. Based on quasi-static analysis of peg–hole insertion operations, Whitney greatly reduced the occurrence of the two problems using a remote compliance centre (RCC) device.

RCC devices are nowadays commercially available tools to facilitate peg–hole assembly operations [13,14]. Zhang *et al.* [8] conducted quasi-static analysis on cylindric peg–hole withdrawal and implemented an active compliance manipulator using a KUKA robot for disassembly purposes. Compliance techniques were developed for jamming avoidance in multiple peg–hole assembly problems [15,16]. In addition to quasi-static peg–hole analysis, dynamic models attracted attention in high-speed scenarios. Asada & Kakumoto [17] analysed the dynamic process of high-speed peg in hole insertion and designed an RCC mechanism for that purpose. Xia *et al.* [18] addressed the limitation of using rigid body dynamics models and investigated jamming and wedging with contact deformation. Vibratory alignment and insertion strategies with RCC devices were adopted in dynamic assembly to avoid jamming and wedging [19,20].

**Figure 1.**
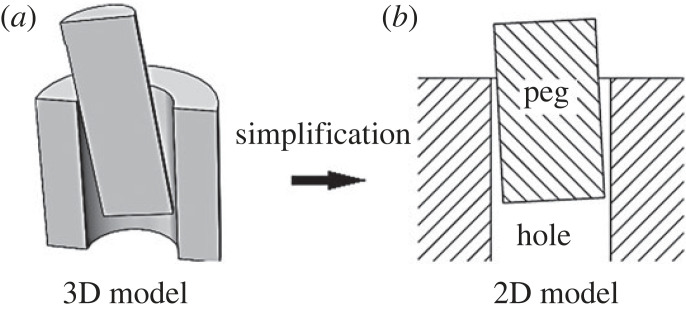
Simplification of peg–hole model from three-dimensional to two-dimensional.

The above surveyed quasi-static and dynamic analyses of the peg–hole problem were based on a common approach: the simplification of the three-dimensional peg–hole model to a two-dimensional representation as in figure 1. This paper examines the correctness of this approach. In detail, the use of a two-dimensional model to represent a three-dimensional peg–hole assembly would be justified if the two below hypotheses held:

(i) The maximum number of contact points between the peg and the wall of the hole is two (if line contact is not considered).
(ii) The forces on the peg lie in a two-dimensional vertical plane including the two contact points.

The above hypotheses define the geometrical and kinematic equivalence of the two representations. Geometrical equivalence means the three-dimensional and two-dimensional models have the same number of contact points, and that these points have the same location. The sufficient and necessary condition for geometrical equivalence is that all the contacts are on a vertical sectional plane (i.e. two-dimensional model), so that the sectional view of the peg–hole system on this plane can fully represent the contact status of the three-dimensional model. Kinematic equivalence means that the forces acting on the peg are the same in the three-dimensional and two-dimensional models. That is, the forces on the peg should lie in the two-dimensional sectional view plane, so that no force has a component perpendicular to the sectional plane. At present, there is no evidence supporting the above conditions for geometrical and kinematic equivalence.

The paper is structured as follows. Section 2 introduces the peg–hole problem. Section 3 discusses the geometrical equivalence of the two-dimensional and three-dimensional representation, and kinematic equivalence is analysed in §4.

## 2. Preliminary analysis

**Figure 2.**
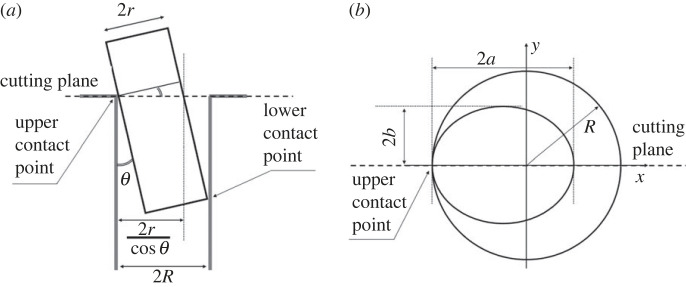
Commonly studied section view of a three-dimensional peg–hole system. The cutting plane is perpendicular to the hole axis of symmetry in (*a*) and includes it in (*b*).

A typical simplification of a three-dimensional peg–hole model into a two-dimensional representation uses the vertical cross-sectional views shown in figure 2. Studies on jamming and wedging problems focus on two-point contacts: one at the bottom of the peg (lower contact point), and the other at the mouth of the hole (upper contact point). That is, it is assumed that the peg is partly inserted in the hole (i.e. the bottom surface is fully below the hole mouth), and its length is larger than the diameter of the hole (i.e. the peg cannot be inserted transversally into the hole).

The radii of the peg and the hole are indicated, respectively, with *r* and *R*, where *r* < *R* (the radius of the peg is smaller than the radius of the hole). The peg has an inclination respect to the hole axis of *θ* as shown in figure 2*a*, where 0 ≤ *θ* < *π*/2 (the case that *θ* ≤ 0 is symmetric respect to the hole axis). In the figure, the axes of the peg and hole lie in the sectional plane.

Figure 2*b* shows the sectional view of a three-dimensional peg–hole model from above, on the hole mouth plane perpendicular to the hole axis of symmetry, where the cross-section of the cylindrical peg projects an ellipse. Most commonly [8,21–23], the point of contact is assumed at the left extreme of the major axis of the ellipse. The semi-major axis length *a* is related to the inclination angle *θ* as in equation (2.1).

2.1 $$a=\frac{r}{\cos\theta }.$$

The semi-minor axis length *b* is the same as the radius *r* of the peg (*b* = *r*). The hole is seen as a circle of radius 2*R*. The circle and ellipse are complete curves, and the ellipse is bound to be inside the circle. The semi-major axis of the ellipse is equal to or smaller than the radius of the circle (*a* ≤ *R*). Substituting *a* ≤ *R* into equation (2.1) yields the following upper bound for the inclination angle:

2.2 $$\theta \leq \text{arccos}\frac{r}{R}.$$

Hence, since *θ* is defined positive,

2.3 $$0<\theta \leq \text{arccos}\frac{r}{R}<\frac{\pi }{2}.$$

**Figure 3.**
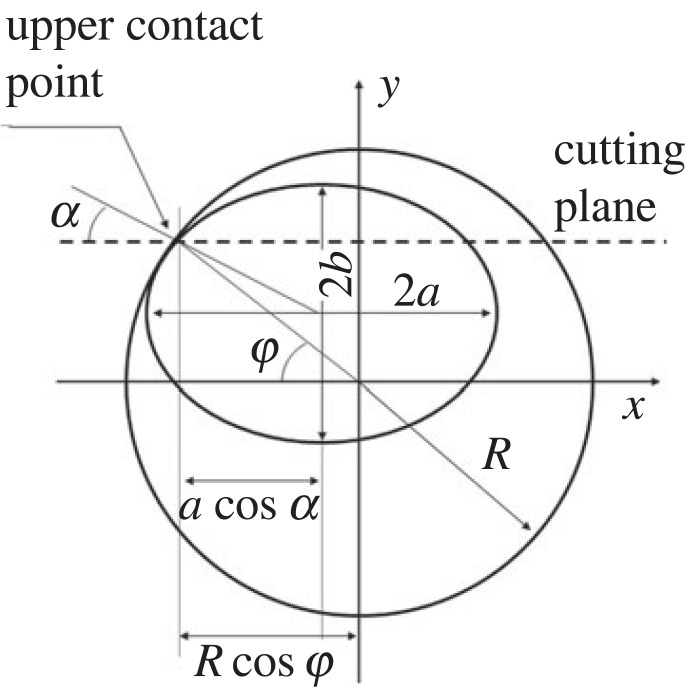
General case of a three-dimensional peg–hole system. The figure should be compared with figure 2, the cutting plane no longer divides the hole into two equal halves.

Equation (2.3) describes the range of *θ*. In the most general case, the point of contact between the peg and the hole is not on the ellipse major axis, and equation (2.2) becomes:

2.4 $$\theta \leq \text{arccos}\frac{r\cos\alpha }{R\cos\phi },$$

where *ϕ* and *α* are as in figure 3. Equation (2.2) is still valid to describe the maximum range of the inclination angle.

**Figure 4.**
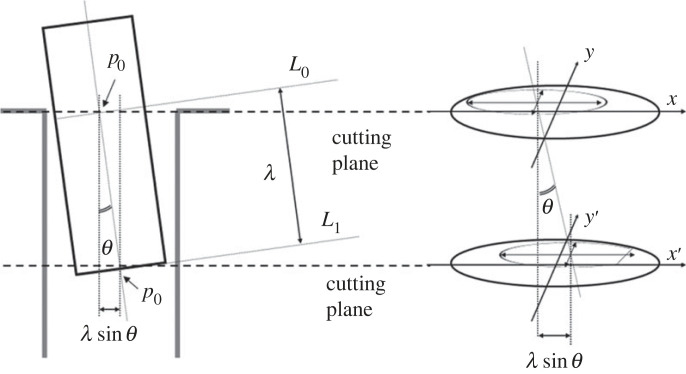
Side view showing the peg insertion length (left) and cutting planes, and corresponding sectional views (right).

Figure 4 shows the peg partly inserted with inclination *θ*, and the two cutting planes at the level of the lower and upper contact point. The peg and hole delineate onto both cutting planes an ellipse and a circle, respectively. In this general case, the ellipse at the lower contact point is not complete.

Now, consider the peg as a line segment and define the ‘zero’ point *p*_0_ as the centre of the peg cross section perpendicular to the peg axis, lying in the hole mouth plane (indicated as *L*_0_ in figure 4). Define the lower extreme *p*_1_ lying at the bottom end of the peg (the centre of the cross section indicated as *L*_1_ in figure 4. The *insertion length* (depth) *λ* is defined as the Euclidean distance between *p*_1_ and *p*_0_:

2.5 $$\lambda =d(p_{1}-p_{0})=\|p_{1}-p_{0}\|.$$

That is, the insertion length describes how deep the peg is inside the hole. Note the peg and hole system can always be oriented so as the peg axis is inclined with an angle *θ* ≥ 0 in a plane parallel to the cutting plane, as shown in figures 2–4.

The geometrical two-dimensional representations in figures 2–4 of the three-dimensional peg–hole system are accurate only if there are indeed only two contact points (one upper and one lower) between the peg and the wall of the hole. The next section will prove the correctness of this assumption, and hence the geometrical equivalence of the two-dimensional and three-dimensional representations.

## 3. Geometrical equivalence

The general case of the peg–hole system is shown in figure 4. Remembering that the peg is assumed to be partly inserted in the hole, it follows that the ellipse drawn by the peg on the sectional plane of the hole mouth must always lie inside the circle drawn by the hole, and that the cross-section of the peg is always a full ellipse. At the point(s) of contact, the ellipse meets the circle. Also, any point of contact lies in the same quadrant of both shapes (e.g. a point of contact in the first quadrant of the circle meets the ellipse at a point in its first quadrant). Finally, if the peg is inclined with a positive *θ* angle as in figure 4, any upper point of contact must lie in the second or third quadrant of the circle drawn by the cross-section of the hole (figure 3). If not, the peg would exit the hole walls below the hole mouth due to its inclination.

To have multiple points of contact, two conditions must apply. Since the ellipse cannot cross the perimeter of the circle, its curvature *κ*_*e*_(*p*) at any point of contact *p* must be higher than the circle’s curvature *κ*_*c*_(*p*) (*Condition A*). If the ellipse and circle have more than one point of contact, the curvature *κ*_*e*_(*p*_1_, *p*_2_) of the arc between any two points of contact *p*_1_ and *p*_2_ of the inner curve (the ellipse) must be smaller than the curvature *κ*_*c*_(*p*_1_, *p*_2_) of the arc of the outer curve (the circle) *Condition B*.

**Figure 5.**
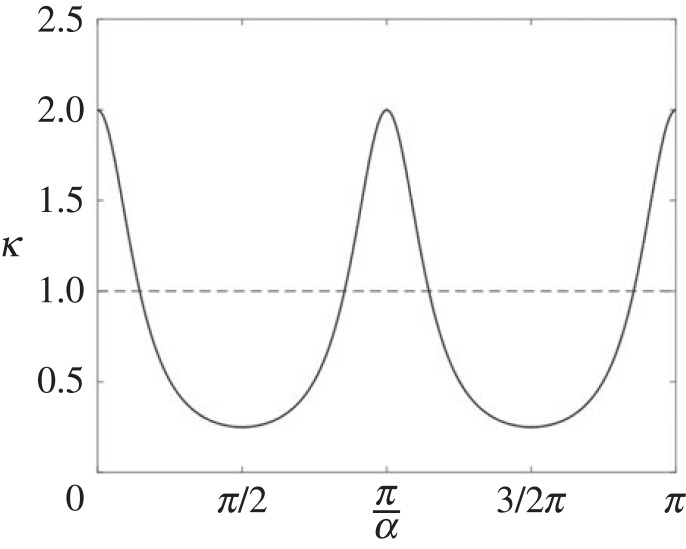
The curvature of a sample ellipse in the [0, 2*π*] interval. The axes of the ellipse are *a* = 2 and *b* = 1. The dashed line shows the curvature of a sample circle of radius *R* = 1.

As shown in figure 5, the ellipse’s curvature *κ*_*e*_ is maximum at the extremes of the major axis (*α* = 0, *π*), minimum at the extremes of the minor axis (*α* = ±*π*/2) and monotonically decreasing or increasing in any of the four quadrants of the shape. The curvature *κ*_*c*_ of the circle is always constant and equal to 1/*R*.

If there were two points of contact *p*_1_ and *p*_2_ in any quadrant of the ellipse, they should both lie in the region where *κ*_*e*_(*p*) > *κ*_*c*_(*p*) (*Condition A*). However, due to the monotonicity of the ellipse’s curvature in one quadrant, *κ*_*e*_(*p*_1_, *p*_2_) > *κ*_*c*_(*p*_1_, *p*_2_) and *Condition B* would not be verified. *Condition B* is not verified also if the two points of contact lie, respectively, before and after the extremes of the major axis (e.g. in the second and third quadrant). In this latter case, both points would lie in the region where *κ*_*e*_(*p*) > *κ*_*c*_(*p*) (*Condition A*), and everywhere in the interval [*p*_1_, *p*_2_] *κ*_*e*_(*p*) > *κ*_*c*_(*p*) (figure 5).

**Figure 6.**
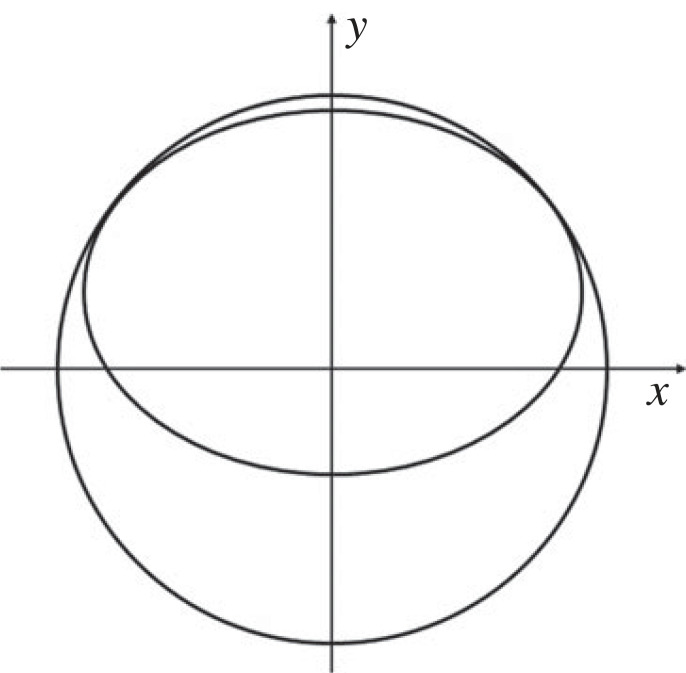
A potential configuration with two upper points of contact.

It follows that the only possible occurrences are two points of contact respectively before and after the extremes of the minor axis, that is one point of contact in the first (fourth) quadrant of the circle, and one in the second (third) quadrant. An example of such configuration is shown in figure 6. However, as remarked before, all upper point of contact must lie in the second or the third quadrant of the circle. Thus, it can be concluded that there cannot be two points of contact, and hence the upper point of contact is unique.

**Figure 7.**
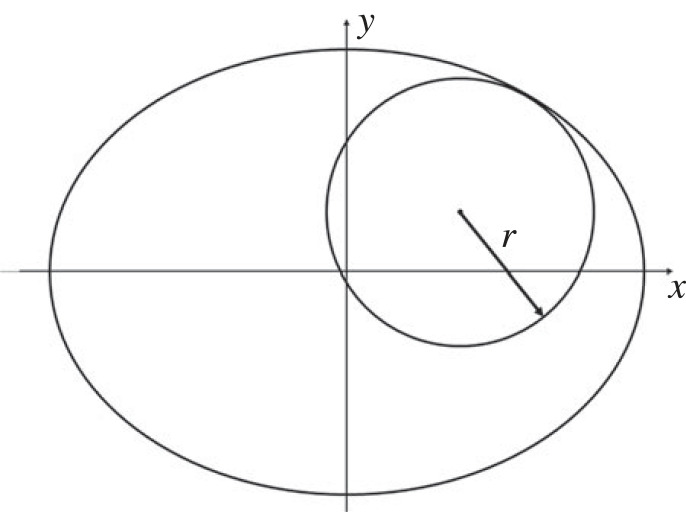
Lower contact point. The sectional plane cuts the peg–hole system at the level of the bottom face of the peg and is perpendicular to the peg axis of symmetry.

To prove that also the lower point of contact is unique, it is useful to change perspective and visualise the peg–hole assembly from a sectional plane perpendicular to the peg axis of symmetry and cut the peg–hole system at the level of the bottom face of the peg. In this case, the cross-section of the peg is a circle, and the cross-section of the hole becomes an ellipse. Figure 7 shows this new sectional view.

Analogously to the case of the upper contact point, if there were two lower contact points *Condition A* and *Condition B* would apply, with the only difference that now the inside shape is the circle and the outside shape is the ellipse. The minimum radius of curvature of the ellipse is:

3.1 $$\rho =\frac{b^{2}}{a}=R^{2}\frac{\cos\theta }{R}=R\cos\theta ,$$

where *b* = *R* is the semi-minor axis and *a* = *R*/cos *θ* the semi-major axis, and 0 < *θ* < *π*/2 is the inclination of the peg. The radius of curvature of the circle is constant and equal to *r*. From equation (2.2):

3.2 $$R\geq \frac{r}{\cos\theta }$$

and hence

3.3 Rcos⁡θ≥r.

That is, the radius of curvature of the ellipse is always greater than the radius of curvature of the circle, and hence the curvature of the ellipse (outside) is always lower than the circle’s (inside). Hence, *Condition B* can never occur.

Therefore, the two-dimensional and three-dimensional representations are geometrically equivalent. There exists a vertical sectional plane passing through the unique upper and lower contact points, where the peg and hole are simplified as rectangles. In this section, it was assumed that the contact point is in the upper half of the circle (hole cross section) in figure 3. Owing to the symmetry of the circle, the considerations made on the uniqueness of the contact point would apply also if the point of contact was in the lower half of the circle. Henceforth, unless explicitly stated, only the case that the point of contact is in the upper half of the circle will be considered. The next section will discuss kinematic equivalence.

## 4. Kinematic equivalence

The analysis of geometrical equivalence implies the feasibility of constructing a two-dimensional representation from a three-dimensional peg–hole model. However, it is still unknown if forces and torques are equivalent in the two-dimensional and three-dimensional models. Kinematic equivalence between the two-dimensional and three-dimensional models defines the viability to simplify the complex three-dimensional forces in the two-dimensional representation without the loss of correctness. Jamming and wedging, the two key issues in peg–hole insertion and withdrawal should be analysed and predicted accurately. Existing analysis on jamming and wedging phenomena in peg–hole model are based on two-dimensional representations [8,12]. This section investigates the kinematic equivalence between two-dimensional and three-dimensional peg–hole models.

**Figure 8.**
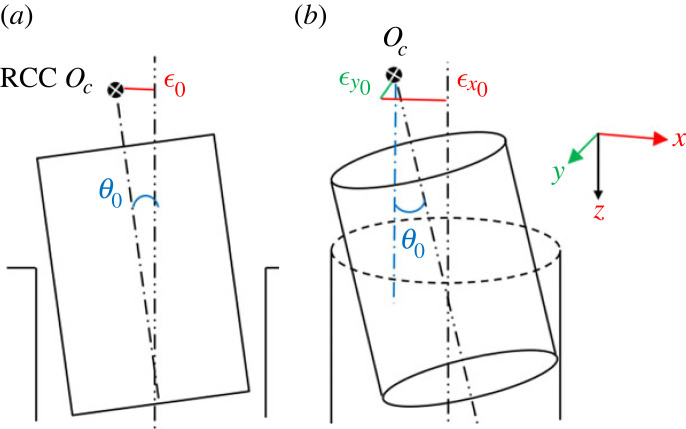
Initial conditions of two-dimensional and three-dimensional peg–hole models with RCC. (*a*) The lateral and angular errors are assumed as co-planar in the two-dimensional representation. (*b*) In the three-dimensional model, the lateral error is not in the two-dimensional sectional plane and is not aligned with the angular error.

In a two-dimensional peg–hole model, the misalignment of the peg and hole is modelled as an angular error *θ*_0_ and a lateral error $\epsilon_{0}$ under the RCC framework. The two errors *θ*_0_ and $\epsilon_{0}$ are ideally co-planar in a two-dimensional sectional plane, as shown in figure 8*a*. Unfortunately, this alignment very rarely occurs in industrial robot assembly and disassembly configurations. Namely, the lateral error consists of two components: $\epsilon_{x_{0}}$ and $\epsilon_{y_{0}}$, the former parallel and the latter perpendicular to the sectional plane (figure 8*b*). In practice, the three-dimensional model has an extra lateral error component in the *y* axis direction, and the upper and lower contact points do not lie in the two-dimensional sectional plane of figure 8*a*.

**Figure 9.**
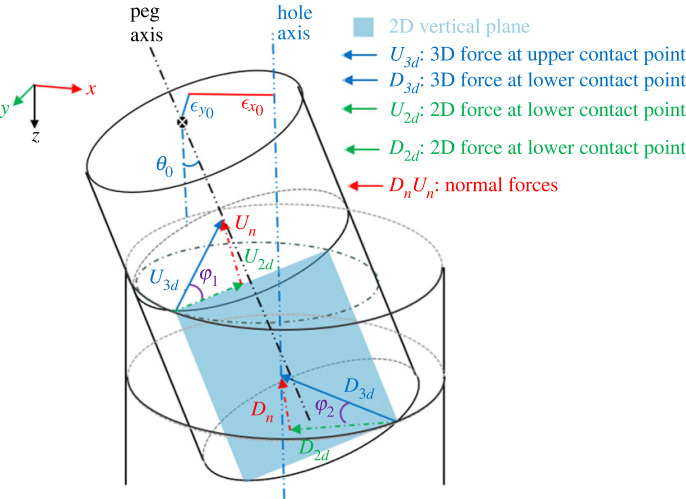
Kinematic properties of the peg–hole model. The sectional view is the same as in figure 3. *U*_3*d*_ and *D*_3*d*_ are the actual three-dimensional forces acting on the peg, and *U*_2*d*_ and *D*_2*d*_ are the forces considered in the two-dimensional cross-sectional model.

Figure 9 shows the three-dimensional contact forces acting on the peg at the upper and lower contact points. The blue area is the two-dimensional vertical plane passing through the two contact points, and generally does not cut the hole mouth in two semicircles. The three-dimensional supporting force *U*_3*d*_ and *D*_3*d*_ at the upper and lower contact points, respectively, points to the peg axis and hole axis. The three-dimensional force *U*_3*d*_ at the upper contact point can be decomposed into two components: a two-dimensional in-plane force *U*_2*d*_ in the two-dimensional vertical plane, and a normal force *U*_*n*_ perpendicular to the two-dimensional vertical plane. The in-plane force *U*_2*d*_ used in the analysis of two-dimensional model is not strictly equal to the actual three-dimensional force *U*_3*d*_, and thus kinematic equivalence is not true. The three-dimensional force *D*_3*d*_ at the lower contact point can be analysed in the same way.

The angles φ_1_ and φ_2_ in figure 9 describe the approximation error between the direction of the actual forces *U*_3*d*_ and *D*_3*d*_ in the three-dimensional model and the direction of the forces *U*_2*d*_ and *D*_2*d*_ used in the two-dimensional peg–hole model. The amplitude of φ_1_ and φ_2_ depends on the geometry (e.g. lateral errors) and kinematic parameters (e.g. the stiffness of the RCC device and the friction coefficients) of the three-dimensional peg–hole model. The stiffness of the RCC device refers to the stiffness coefficients *K*_*x*_ and *K*_*y*_ in [12]. The coefficient of friction refers to the friction coefficient between the peg and the hole. The derivation of the static/quasi-static equations involving stiffness/friction is given in [12]. For a particularly unfavourable configuration of the peg–hole system, the angles φ_1_ and φ_2_ between *F*_2*d*_ and *F*_3*d*_ could be large, leading to a large approximation error between the two-dimensional and three-dimensional models.

The percentage difference between the two-dimensional and three-dimensional analysis depends on the angles φ_1_ and φ_2_ in figure 9, i.e. the percentage difference between the two-dimensional force and three-dimensional force equals 100(1 − cos φ_*i*_), *i* = 1, 2. The greater the angles φ_1_ and φ_2_ are, the larger the percentage difference is. For instance, if the angles φ_1_ and φ_2_ are 0, the two-dimensional and three-dimensional forces are identical; if the angles φ_1_ and φ_2_ approach 90°, the percentage difference is nearly 100%.

Obtaining analytical expressions for φ_1_ and φ_2_ involves solving a set of ellipse and circle equations, for which there may be no solution. For example, to solve the angle φ_2_ in figure 3, let a horizontal sectional plane pass the lower contact point of the peg–hole system, and the tangent point of the peg ellipse and the hole circle is the lower contact point. This tangent point gives the angle φ_2_. This process involves solving a set of equations which of general form is given as follows:

4.1 $$\left\{ \begin{array}{c} {\left(x-x_{h}\right)}^{2}+{\left(y-y_{h}\right)}^{2}=R^{2}\\ \frac{{\left(x\right)}^{2}}{a^{2}}+\frac{{\left(y\right)}^{2}}{b^{2}}=1, \end{array}\right.$$

where the origin of the coordinate system is set as the centre point of the hole circle. (*x*_*p*_, *y*_*p*_) is centre points of the peg ellipse, *R* is the radius of the hole, and *a* and *b* are coefficients of the peg ellipse, respectively. The solution (*x*, *y*) of this equation group gives the angle $\varphi_{2}\;:\;\varphi_{2}=\arctan(y/x)$.

On the other hand, a qualitative analysis of the angles φ_1_ and φ_2_ can still reveal the severity of the approximation error: The maximum allowed angles φ_1_ and φ_2_ range from 0° to 90°. The two extreme cases are explained as follows: (i) 0% difference (φ_1_, φ_2_ = 0°): the peg axis intersects the hole axis, and the two-dimensional forces are identical to the three-dimensional forces. (ii) 100% difference (φ_1_, φ_2_ → 90°): the inclination angle *θ* is nearly 0°, or the insertion length is nearly 0.

### 4.1. Effects on jamming

**Figure 10.**
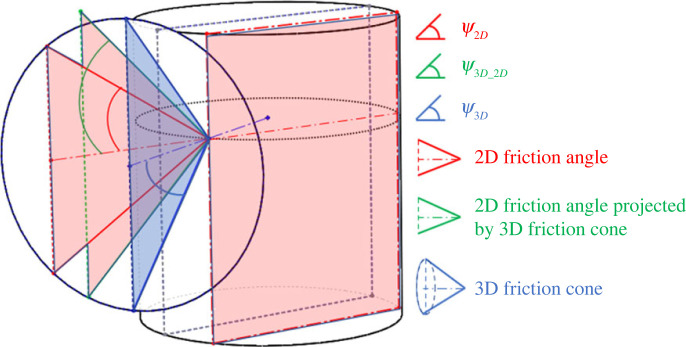
The friction cone at the contact point at the three-dimensional peg. The red and green triangles are co-planar with the red rectangle, that is, they are in a two-dimensional peg hole model. The blue triangle is not co-planar with the two-dimensional plane of the red rectangle.

Jamming is an undesirable phenomenon in the peg–hole system where the peg is unable to move as a consequence of improperly applied forces. Namely, during the insertion/withdrawal process, the assembly/disassembly forces at the contact points are directed inside the friction cone (figure 10).

The forces and moments used in the two-dimensional model are derived by projecting the actual three-dimensional forces on the two-dimensional vertical sectional plane. However, the two-dimensional circular sector drawn by the friction cone is still determined by the frictional coefficient. That is, the angle subtended by the friction cone in the three-dimensional model is equal to the angle subtended by the circular sector in the two-dimensional model. Figure 10 shows the three-dimensional friction cone and two-dimensional circular sector at the upper contact point. The distribution of forces in the three-dimensional model for the lower contact point can be derived similarly. The three-dimensional cone axis is aligned with the supporting force at the upper contact point. The three-dimensional cone angle *ψ*_3*d*_ and the two-dimensional circular sector angle *ψ*_2*d*_ depend on the same frictional coefficient *μ*, thus, *ψ*_3*d*_ = *ψ*_2*d*_ = tan *μ*. The cone angle $\psi_{3d\_2d}$ projected by the three-dimensional cone onto the two-dimensional model is greater than or equal to *ψ*_3*d*_ since the three-dimensional cone axis could be out of the two-dimensional sectional plane. Hence, $\psi_{3d\_2d}\geq \psi_{3d}=\psi_{2d}$.

Jamming occurs if the applied contact force is within the friction cone. The boundary conditions for the jamming phenomenon have been analysed for the two-dimensional peg–hole model in the literature [12]. In the two-dimensional model, the contact force is projected onto the two-dimensional plane. If the direction of the two-dimensional contact force is within the cone, jamming is predicted in this case. However, considering the actual three-dimensional model, the three-dimensional contact force and three-dimensional friction cone are not restricted in the two-dimensional plane. There are two possible cases for three-dimensional model:

— Jamming occurs: The three-dimensional contact force is within the three-dimensional friction cone. However, in the two-dimensional model, only the projected three-dimensional force on the two-dimensional plane is considered and checked against the two-dimensional friction cone. The two-dimensional model would erroneously predict no jamming in the case if the two-dimensional projected force is outside the perimeter of the (red) triangle formed by the two-dimensional friction cone but inside the green triangle in figure 10, that is, *ψ*_2*d*_ < *ψ* < *ψ*_3*d*−2*d*_ where *ψ* is the angle between the contact force and the cone central axis in the two-dimensional model.
— Jamming does not occur: The two-dimensional model will erroneously predict jamming in this case if the three-dimensional contact force is beyond the three-dimensional friction cone in three-dimensional space but the projection of the three-dimensional contact force is inside the two-dimensional (red) friction triangle due to the degenerated nature of the projection.

two-dimensional In summary, the prediction of jamming in the two-dimensional two-dimensional two-dimensional model is based on degenerate data, which only considers the relationships between the contact forces projected on a plane and friction ‘cones’. The modelling of jamming using friction self-locking criteria is not always able to represent the true three-dimensional phenomenon.

### 4.2. Effect on wedging

In the peg–hole model, wedging is the phenomenon where the peg is stuck in the hole and remains so even after the withdrawal of externally applied forces. The forces at the contact points caused by geometrical deformation are self-locked within the friction cone. After removing the externally applied forces, the residual forces are in equilibrium and the peg cannot move any more [12].

**Figure 11.**
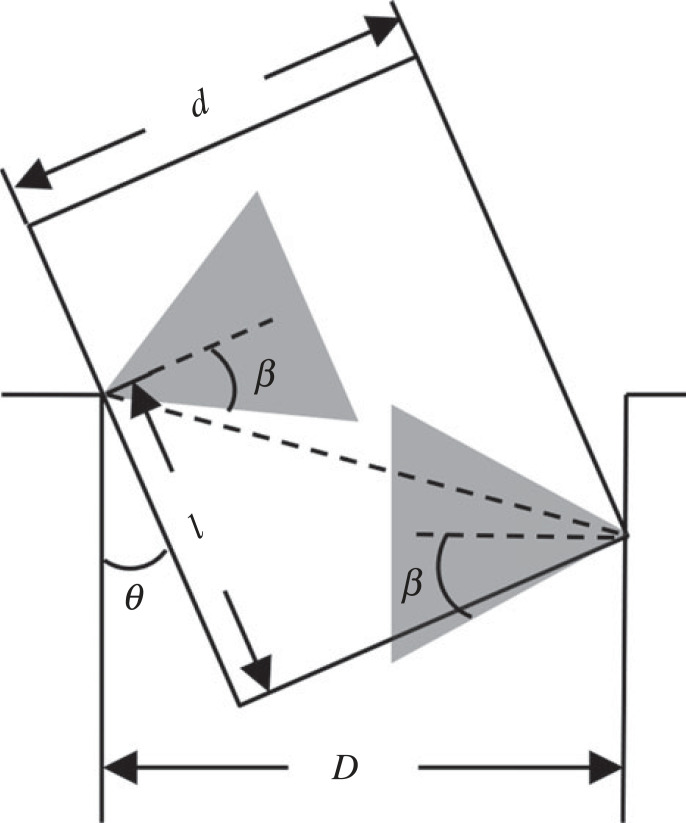
Geometrical conditions of wedging in the two-dimensional peg–hole model.

Figure 11 shows the geometrical conditions for wedging in a two-dimensional peg–hole model. The shaded triangles are frictional triangles calculated using the frictional coefficient β=arctan⁡μ, where *μ* is the frictional coefficient. The necessary condition for wedging has been demonstrated to be that the two friction cones overlap, namely that *l* ≤ *l*_*w*_ = *μd* where *l*_*w*_ is the upper bound on the insertion length of the peg. This boundary condition can also be formulated using the minimum inclination angle *θ*_*w*_, that is, *θ* ≥ *θ*_*w*_ = *c*/*μ*, where *c* is the clearance ratio *c* = (*D* − *d*)/*D* (*θ*, *D*, *d* as in figure 11).

In a three-dimensional peg–hole model, the boundary conditions are narrowed due to the extra lateral error as discussed in the previous section (figure 8). Owing to the extra lateral error $\epsilon_{0}$, the analysis of the wedging condition is affected by the following two factors.

**Figure 12.**
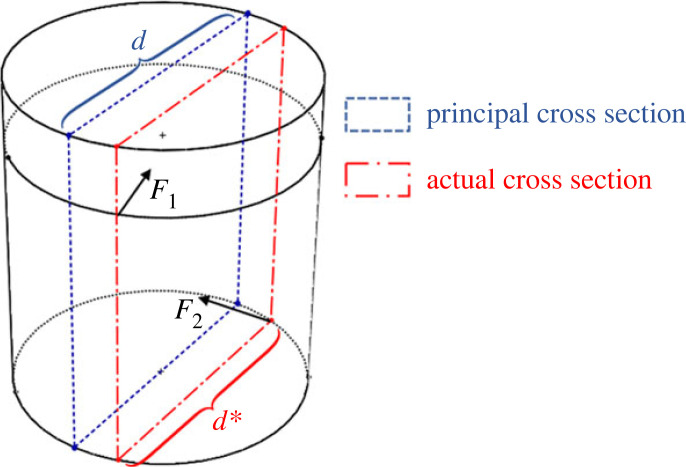
The actual sectional plane is shifted due to the extra lateral error.

First, the extra lateral error reduces the peg diameter. In the two-dimensional peg–hole model, the peg diameter *d* used to determine the wedging boundary condition is based on the principal cross section passing through the peg axis as shown in figure 12. In a three-dimensional model, the extra lateral error shifts the actual sectional plane beyond the principal cross section. The peg diameter d* for the actual cross section is thus smaller than the principal cross section, i.e. d* < *d*.

**Figure 13.**
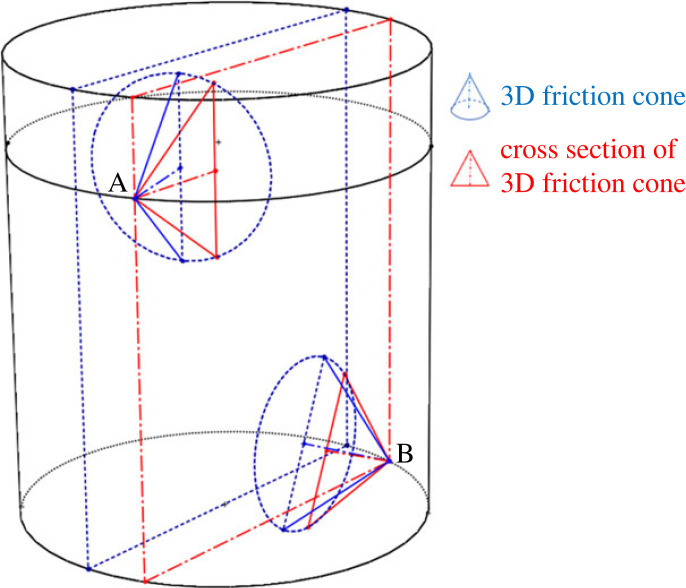
The two-dimensional and three-dimensional friction cone on the peg.

Second, the extra lateral error in the three-dimensional model changes the orientation of the friction cones. In the two-dimensional model, the friction cone at the contact point is simplified and its axis is considered to lie in the two-dimensional sectional plane. Thus, the in-plane friction angle *β* derives directly from the frictional coefficient *μ*: *β* = tan *μ*. In the three-dimensional peg–hole model, the extra lateral error changes the friction cone axis, moving it beyond the two-dimensional sectional plane. As shown in figure 13, the three-dimensional friction cone axis is not aligned with the two-dimensional sectional plane. Consequently, the in-plane friction cone is shrunk, and the possibility of wedging could be misjudged. The in-plane friction angle β*three-dimensional in the model is decreased by the extra lateral error, i.e. β* ≤ *β* = tan *μ*.

In summary, the extra lateral error in the three-dimensional model shrinks the in-plane peg width and friction angle discussed in the two-dimensional model. The actual maximum insertion length for wedging l* is less than or equal to the insertion length that would be predicted using the two-dimensional model. That is, the extra error in three-dimensional model reduces the area where wedging can occur.

## 5. Conclusion

Understanding peg–hole assembly is important in the study of robotic assembly and disassembly systems. A two-dimensional representation is widely used as an approximation of the actual three-dimensional peg–hole model in the literature, without investigating the approximation error. This paper discussed the geometrical and kinematic equivalence between the full three-dimensional and the two-dimensional simplified model of a peg–hole system. The geometrical equivalence was verified, showing there can be only one upper contact point and one lower contact point. The two-dimensional model can be built by selecting a unique vertical plane passing through these two contact points. However, the forces acting on the peg are not co-planar to the sectional view plane, and this was shown to imply there is no kinematic equivalence between the three-dimensional and two-dimensional representations. The severity of the error introduced by the two-dimensional simplification was shown to depend on the position of the contact point between the peg and the hole. The effects of this approximation error on peg–hole jamming and wedging using RCC mechanisms were discussed by qualitative analysis in this paper. Owing to the complexity of deriving the analytical functions and boundary conditions for jamming and wedging phenomena, this was not done and is left as a topic for future investigation.

## Data Availability

This work did not require ethical approval from a human subject or animal welfare committee.

## Data accessibility

This article has no additional data.
